# Dithiocarbazate Ligand-Based Cu(II), Ni(II), and Zn(II) Complexes: Synthesis, Structural Investigations, Cytotoxicity, DNA Binding, and Molecular Docking Studies

**DOI:** 10.1155/2022/2004052

**Published:** 2022-07-31

**Authors:** Enis Nadia Md Yusof, Mohammad Azam, Siti Syaida Sirat, Thahira B. S. A. Ravoof, Alister J. Page, Abhi Veerakumarasivam, Thiruventhan Karunakaran, Mohd Rizal Razali

**Affiliations:** ^1^Chemistry Section, School of Distance Education, Universiti Sains Malaysia, 11800 Minden, Pulau Pinang, Malaysia; ^2^Department of Chemistry, College of Science, King Saud University, P.O. Box 2455, Riyadh 11451, Saudi Arabia; ^3^Faculty of Applied Sciences, Universiti Teknologi MARA, Cawangan Negeri Sembilan, Kampus Kuala Pilah, Kuala Pilah 72000, Negeri Sembilan, Malaysia; ^4^Department of Chemistry, Faculty of Science, Universiti Putra Malaysia, 43400 UPM Serdang, Seri Kembangan, Selangor, Malaysia; ^5^Discipline of Chemistry, School of Environmental and Life Sciences, University of Newcastle, University Drive, Callaghan, Newcastle, NSW 2308, Australia; ^6^Department of Biological Sciences, School of Medical and Life Sciences, Sunway University, No. 5 Jalan Universiti, 47500 Bandar Sunway, Petaling Jaya, Selangor Darul Ehsan, Malaysia; ^7^Medical Genetics Laboratory, Faculty of Medicine and Health Sciences, Universiti Putra Malaysia, 43400 UPM Serdang, Seri Kembangan, Selangor Darul Ehsan, Malaysia; ^8^Centre for Drug Research, Universiti Sains Malaysia, 11800 Minden, Pulau Pinang, Malaysia; ^9^School of Chemical Sciences, Universiti Sains Malaysia, 11800 Minden, Pulau Pinang, Malaysia

## Abstract

S-4-methylbenzyl-*β*-N-(2-methoxybenzylmethylene)dithiocarbazate ligand, 1, prepared from S-(4-methylbenzyl)dithiocarbazate, was used to produce a novel series of transition metal complexes of the type, [M (L)_2_] [M = Cu(II) (**2**), Ni(II) (**3**), and Zn(II) (**4**), L = **1**]. The ligand and its complexes were investigated by elemental analysis, FTIR, ^1^H and ^13^C-NMR, MS spectrometry, and molar conductivity. In addition, single X-ray crystallography was also performed for ligand, **1**, and complex **3**. The Hirshfeld surface analyses were also performed to know about various bonding interactions in the ligand, **1**, and complex **3**. The investigated compounds were also tested to evaluate their cytotoxic behaviour. However, complex **2** showed promising results against MCF-7 and MDA-MB-213 cancer cell lines. Furthermore, the interaction of CT-DNA with ligand, **1,** and complex **2** was also studied using the electronic absorption method, revealing that the compounds have potential DNA-binding ability via hydrogen bonding and hydrophobic and van der Waals interactions. A molecular docking study of complex **2** was also carried out, which revealed that free binding free energy value was −7.39 kcal mol^−1^.

## 1. Introduction

Coordination compounds have a long history of use as chemotherapeutic medicines and have advantages over organic compounds. The bioinorganic mechanism and standard pharmacokinetic parameters of uptake, distribution, and excretion are used to assess the potential of coordination compounds as effective anticancer agents (as drugs or modifiers of biological response) [1]. Furthermore, medicinal chemists can investigate the potential of metal-containing compounds using a variety of strategies, such as coordination compounds with different coordination numbers, geometries, oxidation states, and ligand substitution thermokinetics [2, 3]. Cisplatin (cis-diamminedichloroplatinum(II)), a platinum-based bifunctional reagent, is an extremely effective chemotherapeutic drug used to treat a variety of malignancies. However, despite their excellent efficacy, these drugs are only used in limited circumstances due to their toxicity, side effects, and acquired resistance in patients [4]. Therefore, to improve therapeutic effectiveness and avoid drawbacks, there is a need for novel platinum and non-platinum-based compounds with potential chemotherapeutic properties [5].

Copper, nickel, and zinc are bio-essential elements and are required for performing various biological functions in the human body. In the field of non-platinum compounds with anticancer properties, complexes with copper, nickel, and zinc have provided a promising alternative to platinum in the development of anticancer drugs, exhibiting activity on cisplatin inactive tumours and the potential to be successful against platinum-resistant malignancies in patients. In addition, copper is also essential for the activity of cytochrome oxidase, superoxide dismutase, ascorbate oxidase, and tyrosinase, enzymes, and proteins involved in energy metabolism, respiration, and DNA synthesis [6]. Furthermore, these metal complexes are likely to have different modes of action, bio-distribution, and toxicity than platinum-based medicines. In addition, the design of ligands associated with metal ions has a considerable impact on drug transport to the target position [7, 8]. Schiff bases, also known as “privileged ligands,” have a characteristic imine linkage and are frequently used as pharmacophores for drug design because of their easy synthesis and high solubility in organic solvents. Furthermore, Schiff bases exhibit significant biological activity owing to the interaction of azomethine nitrogen with protein amino acid residues or DNA nucleobases [9–11].

**Figure 1:**
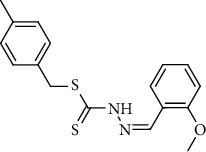
Structure of ligand **1**.

Over the past several years, dithiocarbazate Schiff bases have gained considerable interest because of their promising bioactivities against various cancer cells [12–15]. However, the biological applications of these compounds become changed by the addition of different organic substituents, resulting in even minor structural modifications [12, 16–18]. Furthermore, dithiocarbazate Schiff bases coordinate with the metal ions to form a wide range of metal complexes with numerous biological applications [12, 13, 15–18]. Therefore, considering the diverse significance of dithiocarbazate Schiff bases and their role in a variety of biological applications, herein we are interested to design a novel dithiocarbazate Schiff base ligand (Figure 1) and its complexes with Cu(II), Ni(II), and Zn(II) ions. The ligand and its complexes are characterized by various spectroscopic methods and single-crystal X-ray crystallography in the case of ligand and complex **2**. All the synthesized compounds have been tested to evaluate anticancer activity against MCF-7 and MDA-MB-213 cancer cell lines. Single-crystal X-ray diffraction and Hirshfeld surface studies were used to better define the geometric structure of **1** and **3**. The experimental data were investigated using density functional theory (DFT), and the shape of complex **2** was optimized for molecular docking simulations using DFT.

## 2. Experiments

### 2.1. Materials and Instrumentation

All chemicals and solvents were used without any purification. Chemicals used are as follows: 2-methoxybenzaldehyde (Merck), copper(II) acetate (Univar), nickel(II) acetate tetrahydrate (Fluka), zinc(II) acetate dihydrate (Fluka), and nitric acid (65%) (Fisher). Solvents used are as follows: acetonitrile (Baker), ethanol (99.8%, Scharlau), methanol (Fisher), and dimethyl sulfoxide (Scharlau).

An electrothermal digital melting point equipment was used to measure the melting points, whereas the magnetic susceptibilities were determined at 25°C using a Sherwood Scientific MSB-AUTO magnetic susceptibility balance. A JENWAY 4310 conductivity meter was used to determine the molar conductivities of the complexes in DMSO at 10^−3^ M at 27°C. PerkinElmer Spectrum 100 with universal ATR polarization was used to measure FTIR spectra at 4000–280 cm^−1^. A LECO CHNS-932 analyzer was used to perform analyses of C, H, and N. A Perkin–Elmer Plasma 1000 Emission Spectrometer was used to do metal measurements. Shimadzu UV-1650PC UV-Visible Spectrophotometer was used to measure the electronic spectra at 1000–200 nm. An NMR JNM ECA400 spectrometer was used to record the ^1^H and ^13^C NMR spectra of **1**, while those of **3** and **4** were determined by a Bruker Ascend™ 700 MHz spectrometer. A Shimadzu GC-MS QP2010 plus mass spectrometer was used to collect the mass spectra.

### 2.2. Synthesis of S-4-Methylbenzyl-*β*-N-(2-Methoxybenzylmethylene)dithiocarbazate Ligand (**1**)

**Scheme 1:**
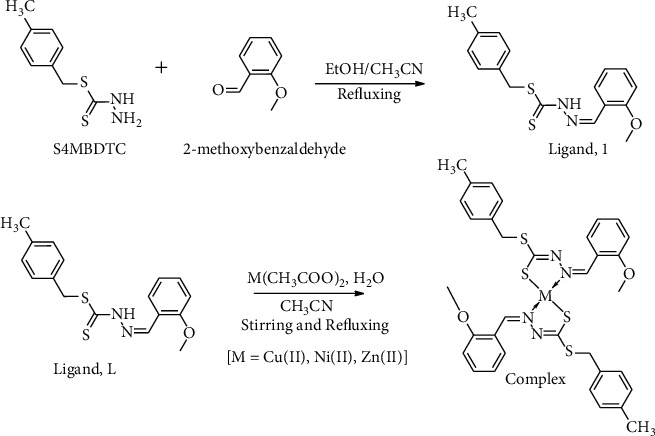
Schematic representation of the ligand, **1,** and its Cu (II), Ni (II), and Zn (II) complexes.

The dithiocarbazate derivative, S-(4-methylbenzyl)dithiocarbazate (S4MBDTC), was synthesized using a reported procedure [12, 18, 19]. S4MBDTC (2.12 g, 0.01 mol) was dissolved (100 mL) in the mixture of hot ethanol and acetonitrile (Scheme 1). An equimolar amount of 2-methoxybenzaldehyde was added to the hot dithiocarbazate solution and refluxed at 70°C for 5 h and then stirred for another 5 h until a precipitate is formed at room temperature, which was then filtered and dried over silica gel, yielding yellow crystals on recrystallization in acetonitrile.

Yield: 81%. Melting point: 167–169°C. Anal. Cal.: C, 61.79; H, 5.49; and N, 8.48%. Found: C, 62.11; H, 5.40; and N, 9.09%. FTIR (ATR, cm^−1^): 3105 *v*_(N − H)_, 1600 *v*_(C=N)_, 1153 *v*_(N − N)_, and 948 *v*_(C=S)_. ^1^H NMR (DMSO-*d*_6_) *δ* (ppm): 2.24 (s, 3H, CH_3_), 3.81 (s, 3H, O-CH_3_), 4.38 (s, 2H, CH_2_), 6.94–7.71 (m, 8H, aromatic-H), 8.55 (s, 1H, CH), and 13.27 (s, 1H, NH). ^13^C NMR (DMSO-*d*_6_) *δ* (ppm): 21.2 (CH_3_), 38.0 (CH_2_), 56.3 (O-CH_3_), 112.6, 121.4, 121.8, 126.0, 129.6, 129.7, 133.0, 134.1, 137.0, 142.7 (aromatic-C), 158.9 (C = N), and 196.8 (S-C = S). *m/z* cal: 330.47 g/mol and found: 330.

### 2.3. General Method for Synthesizing Complexes, [M (**L**)_2_] [M = Cu(II) (**2**), Ni(II) (**3**), and Zn(II) (**4**)]

The hot acetonitrile solution of ligand, **1** (0.66 g, 0.002 mol), was combined with an alcoholic solution of M (CH_3_COO)_2_.nH2O [M = (CH_3_COO)_2_Cu.H_2_O, (CH_3_COO)_2_Ni.4H_2_O, and (CH_3_COO)_2_Zn.2H_2_O] in 1:1 molar ratio (Scheme 1). The reaction mixture was stirred and refluxed at 70°C for 3 hours, which was subsequently allowed to cool at room temperature, resulting in the formation of a precipitate. The precipitate was separated and purified by drying it over silica gel, followed by recrystallization in methanol. We were successful in obtaining single crystals for **3**. However, despite our best efforts, we failed to grow single crystals suitable for single-crystal X-ray diffraction for **2** and **4**.

  Copper(II) Complex (**2**).Green powder. Yield: 80%. M.p.: 180–182°C. Anal. Cal.: C, 56.52; H, 4.74; N, 7.75; and Cu, 8.80%. Found: C, 56.59; H, 4.45; N, 8.01; and Cu, 8.53%. FTIR (ATR, cm^−1^): 1596 *v*(C=N), 1100 *v*(N − N), and 943 *v*(C=S).
  Nickel(II) Complex (**3**). Brown crystals. Yield: 83%. M.p.: 182–183°C. Anal. Cal.: C, 56.91; H, 4.78; N, 7.81; and Ni, 8.18%. Found: C, 56.47; H, 4.72; N, 8.57; and Ni, 8.39%. FTIR (ATR, cm^−1^): 1589 *v*(C=N), 1107 *v*(N − N), and 964 *v*(C=S). ^1^H NMR (CDCl_3_) *δ* (ppm): 2.35 (CH_3_), 3.78 (s, 6H, O-CH_3_), 3.88 (s, 4H, CH_2_), 7.04–7.75 (m, 16H, aromatic-H), and 8.40 (s, 2H, CH). ^13^C NMR (DMSO-*d*_*6*_) *δ* (ppm): 21.2 (CH_3_), 39.6 (CH_2_), 55.4 (O-CH_3_), 112.4, 117.2, 117.6, 119.1, 121.8, 126.0, 128.9, 129.3, 129.4, 132.8, 133.1, 135.3 (Ar-C), 159.4, 159.8 (C = N), and 168.4 (S-C-S).
  Zinc(II) Complex (**4**). Yellow powder. Yield: 83%. M.p.: 197–198°C. Anal. Cal.: C, 56.38; H, 4.73; N, 7.74; and Zn, 9.03%. Found: C, 56.53; H, 4.70; N, 8.48; and Zn, 9.23%. FTIR (ATR, cm^−1^): 1585 *v*(C=N), 1100 *v*(N − N), and 944 *v*(C=S).^1^H NMR (CDCl3) *δ* (ppm): 2.28 (CH_3_), 3.70 (s, 6H, O-CH_3_), 3.82 (s, 4H, CH_2_), 6.67–7.61 (m, 16H, Ar-H), and 8.83 (s, 2H, CH). ^13^C NMR (DMSO-*d*_*6*_) *δ* (ppm): 21.4 (CH_3_), 36.6 (CH_2_), 55.7 (O-CH_3_), 111.0, 120.1, 120.9, 121.1, 129.1, 129.3, 129.4, 134.2, 134.8, 136.9 (Ar-C), 159.4 (C = N), and 179.4 (S-C-S).

### 2.4. Single-Crystal X-Ray Structure Determination

The intensity data for the single crystal of the ligand, 1, were obtained on a CrysAlisPro Oxford diffractometer fitted with graphite monochromated Cu-K radiation (*λ* = 1.54 Å), at 296 K, whereas the measurements for complex 3 were processed on a CCD area detector APEXII Duo diffractometer operating at 50 kV and 30 mA with Mo-K*α* radiation (*λ* = 0.71073 Å) at 296 K.

SAINT and APEXII software packages were used to integrate the data, and the SADABS tool was used to perform the empirical absorption correction [20]. Direct methods using SHELXS-2014 were applied to find the solution and subsequently refined by applying the full-matrix least-squares method to F2 using SHELXL-2014 [21]. X-Seed was used as a graphic interface for SHELXL [22]. Non-hydrogen atoms were refined anisotropically, while the refinement for hydrogen atoms was isotropic.  Table 1 shows the entire crystal data and refinement details.

### 2.5. Density Functional Theory (DFT) Calculation

Gaussian09 [23] and GaussView5 [24] software packages were used to conduct DFT calculations. The DFT approach was used to fully optimize the structure of complexes **2** and **4** utilizing the B3LYP [25, 26] hybrid exchange correlation functional with LanL2DZ pseudopotential on Cu and Zn [27–29] and the 6-311G (d, p) Pople basis set for all other atoms. The initial structures and geometries of **1** and **3** were obtained from single-crystal X-ray diffraction analysis. The DFT calculation for **1** and **3** was analysed with the same functional and basis sets. A scaling factor of 0.9682 was used to scale vibrational frequencies [30]. The HOMO-LUMO energies of the optimized geometries were calculated with time-dependent DFT (TD-DFT) measurements on the same basis set [31, 32], and the solvation effects (DMSO) were added using the polarizable continuum method (PCM) [33–35].

### 2.6. Hirshfeld Surface Analysis

The Hirshfeld surfaces and 2D fingerprint plots were generated using CrystalExplorer 17.5 [36]. The structural input files for **1** and **3** were obtained in the CIF format. A normalized contact, denoted by d_norm_, is a ratio that takes into account the distances between any surface point and the closest internal (*d*_i_) and exterior (*d*_e_) atoms, as well as the van der Waals radii of the atoms.

### 2.7. Cell Culture and Viability Assay

ATTC, Virginia, USA, provided human breast cancer cells with both estrogen-positive (MCF-7) and triple-negative estrogen (MDA-MB-231) receptors. Cells were grown at 37°C in RPMI-1640 (high-glucose) media with 10% fetal bovine serum comprising 1% penicillin in a moistened environment of 5% CO_2_ in air. The 3-(4,5-dimethylthiazol-2-yl)-2,5-diphenyltetrazolium bromide (MTT) assay was used to assess the inhibitory effect of the ligand, **1**, and its complexes **2-4** on the proliferation of breast cancer cells [12, 18, 37]. It was observed that ligand, **1**, and its complexes **2-4** were nontoxic to cancer cells when they were dispersed in DMSO and diluted in culture medium to a final DMSO concentration of less than 0.05% (v/v). Cells were sown in 96-well plates at a density of 6000 cells per well for 24 hours before being exposed to various doses of chemicals for 72 hours. The wells were rinsed with 200 *μ*L of phosphate buffer saline after the medium was removed. Each well was filled with aliquots of 20 *μ*L of 3-(4,5-dimethylthiazol-2-yl)-2,5-diphenyltetrazolium bromide (MTT) and incubated at 37°C for 4 hours. Following that, each well that had 2 *μ*L of the medium was replaced with 200 *μ*L of DMSO. An ELISA plate reader was used to determine optical density at 570 nm. In all of the experiments, there were control wells (100% viability) filled with medium and DMSO. The average of triplicate assays was used to generate all of the data points. GI_50_ was used to measure cytotoxicity, which was defined as the concentration that reduced the absorbance of treated cells by 50% when compared to the control (untreated cells) [38].

### 2.8. DNA-Binding Studies via UV-Visible Absorption Spectroscopy

UV-Vis absorption studies were carried out to ensure the CT-DNA interaction of the ligand, **1**, and complex **2** at room temperature. Throughout the absorption titrations, the concentration of the bioactive complex **2** solutions was kept constant at 50 *μ*M, while the concentration of CT-DNA was gradually increased (4.21 × 10^−5^ M). At room temperature, the complex was dispersed in DMSO and diluted in Tris-HCI buffer [39], with wavelength measured at 230 to 600 nm. The absorbance values were taken ten minutes after the DNA solution was injected. The following equation was used to calculate the binding constant, *K*_b_:

(1) $$\begin{array}{c} \frac{\left[\text{DNA}\right]}{\left(\varepsilon_{\text{a}}-\varepsilon_{\text{f}}\right)}=\frac{\left[\text{DNA}\right]}{\left(\varepsilon_{\text{b}}-\varepsilon_{\text{f}}\right)}+\frac{1}{K_{\text{b}}\left(\varepsilon_{\text{b}}-\varepsilon_{\text{f}}\right)}, \end{array}$$

where [DNA] denotes the base-pair concentration, *ε*_a_ is the apparent molar extinction coefficient A_abs_/[M], and *ε*_f_ is the extinction coefficient for free complex **2** [M], while *ε*_b_ is the extinction coefficient for the completely bound complex **2**. The DNA-binding absorption studies followed the same procedure as those described in our earlier publications [19].

### 2.9. Molecular Docking

The Protein Data Bank [40] provided the coordinates for dodecamer d (CGCGAATTCGCG)_2_ of B-DNA (PDB ID : 1BNA). The coordinates of **2** were determined after utilizing the DFT approach to minimize energy. The AutoDock Tools version 1.5.6 and 4.2.5.1 programs were used for molecular docking investigations [41]. Water molecules were removed from the receptor and replaced with polar hydrogen atoms and Kollman charges (DNA sequences). The AutoDock parameter file was updated to include van der Waals interactions and other **2**-specific parameters retrieved from the AutoDock website [42, 43]. The conformation with the lowest binding energy from the highest cluster was chosen as the best ligand-receptor binding conformation [19, 44].

## 3. Results and Discussion

The synthesis of dithiocarbazate Schiff base, **1**, and its complexes with Cu(II), Ni(II), and Zn(II) ions is shown in Scheme 1. The isolated complexes were obtained in good yield (70–83%) and were soluble in DMSO, DMF, acetone, and chloroform at room temperature. The molar conductance values for the isolated complexes at 0.30–2.04 Ω^−1^ cm^2^ mol^−1^ suggested the complexes to be non-electrolytic [45].

### 3.1. Spectroscopic Studies

The IR spectra for the ligand, **1,** and its complexes **2-4** were determined at 400–4000 cm^−1^ (Supplementary Information Table S1 and Figures S1–S4). The IR spectra of the ligand, **1,** exhibit the characteristic bands due to *v*_(NH)_ at 3086 and 3105 cm^−1^ [46, 47]. However, the stretching bands due to *v*_(NH)_ disappeared when ligand, **1**, is complexed with metal ions, suggesting its deprotonation [46, 47]. Furthermore, the strong band appearing at 1600–1595 cm^−1^ in the spectra of the ligand is assigned to *v*_(C=N)_ vibration [46–52], which, however, shifted to lower wavenumber, suggesting the complexation of **1** to metal(II) ions via azomethine nitrogen atoms [46–52]. However, the same pattern was observed for the hydrazine *v*_(N − N)_ band in the spectra of metal(II) complexes, suggesting the coordination of the ligand, **1**, via the nitrogen lone pair, where the electron delocalization occurs through the 5-membered chelate ring [46, 47]. The disappearance of vibrations due to *v*_(C=S)_ and the appearance of the splitting *v*_(CSS)_ bands in the spectra of complexes provide strong evidence that the ligand has been coordinated to metal ion through the thiolate sulphur [49, 53, 54].

The ^1^H NMR spectrum of **1** did not show any signal at ca. *δ* 4.00 ppm, attributed to S-H proton, confirming that **1** exists as thione tautomer even in solution [55]. The occurrence of a proton connected to the nitrogen atom is shown by a singlet signal at 13.29 ppm, which, however, was absent in the spectra of complexes **3** and **4**, indicating the coordination of the Schiff bases to the metal ion. The ^13^C NMR spectrum of **1** showed a downfield chemical shift at *δ* ∼ 196 ppm, indicating the existence of the C = S thione tautomer in solution. In the spectra of complexes **3** and **4**, there was an upfield shift of this signal, suggesting a drop in electron density at the carbon atom as a result of the sulphur atom complexing with metal ion, confirming the structures suggested for the complexes [46–52] (Supplementary Information Figures S5–S10).

The MS spectral data revealed that the molecular ion peak identified in the spectra matches the proposed structure of the ligand, **1**. The mass spectra revealed the presence of a molecular ion peak [C_17_H_18_N_2_OS_2_]^+^ at m/*z* 330, which correspond to the presence of [C_16_H_16_N_2_OS_2_]^+^ and [C_17_H_18_N_2_OS_2_]^+^ molecular ions (Supplementary Information Figure S11).

**Figure 2:**
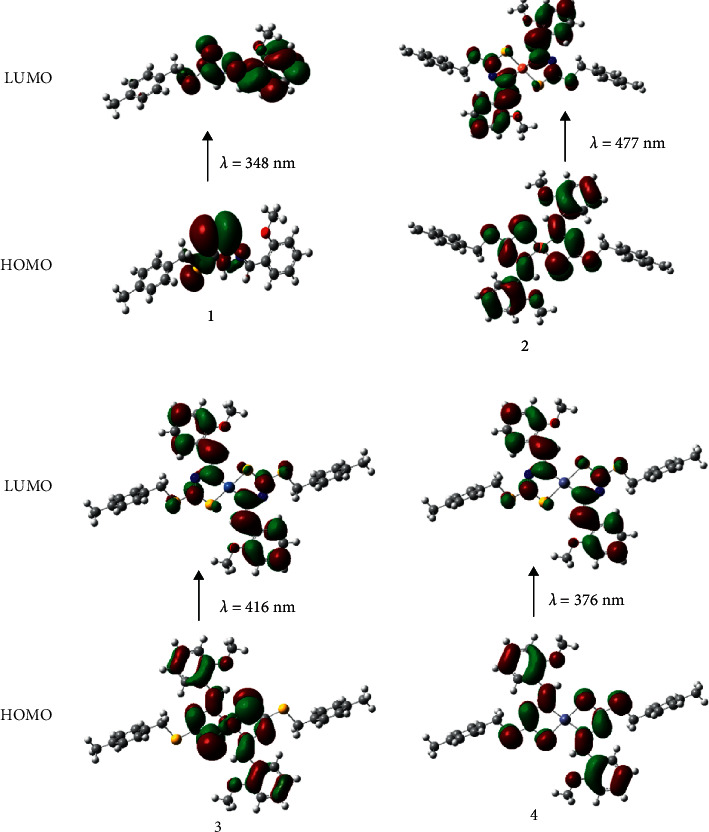
Frontier MOs of the Schiff base **1** and its complexes (**2**–**4**).

The UV-Vis spectra of **1** in DMSO at 10^−3^ M showed a high-intensity peak at 356 nm, which was assigned to *n*⟶*π*^*∗*^ and *π*⟶*π*^*∗*^ transitions and agrees well with the TD-DFT electron excitation at 348 nm [56]. This excitation of electrons occurs at lone pair of nitrogen atoms and aromatic phenyl ring (Figure 2). For **2**–**4**, the HOMO is mainly located on the 3-methoxybenzyl backbone of each dithiocarbazate Schiff bases and the metal centre, whereas the LUMO mainly resides on 3-methoxybenzyl and backbone of dithiocarbazate Schiff bases.  Table S2 contains the UV/Vis data for all compounds.

### 3.2. X-Ray Structure Crystallography

**Figure 3:**
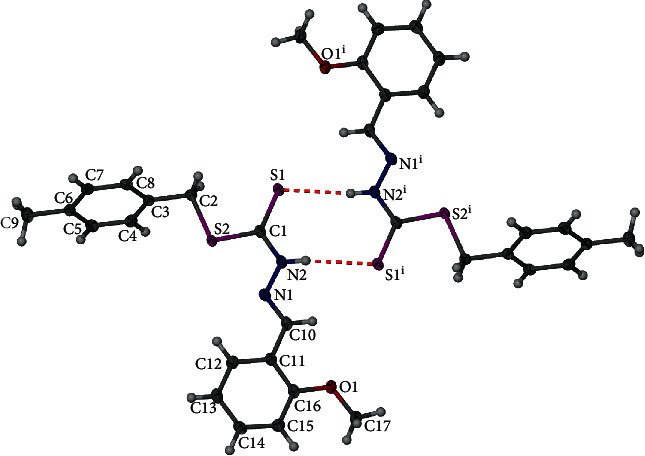
Structure of **1**. Symmetry elements used: (a) ^i^ = −*x*, 2−*y*, −*z, and* (b) ^i^ = 2−*x*, 1−*y*, 2−*z*.

Single-crystal X-ray diffraction data revealed the structures of the ligand, **1**, and its complex **3**. The selected bond lengths and bond angles of **1** and **3** are presented in Table 2. **1** crystallizes in monoclinic space group *P*2_1_/*n* with the whole molecule arranged in an asymmetric unit. In **1**, the dithiocarbazate backbone (C2˗S2˗C1˗S1˗N2) and (N1˗C10˗C11˗C12˗ C13˗C14˗ C15˗C16˗O1˗C17) moiety could be considered that almost coplanar conformation with dihedral angle is shown at 7.19 (5)°. The bond lengths of S2˗C1 = 1.7564 (17) Å, S1˗C1 = 1.6743 (18) Å, N2˗C1 = 1.335 (2) Å, and *N*2˗N1 = 1.376 (2) Å are fall in expected value and almost similar to the those reported literature [53, 57]. The ligand, **1,** behaves similarly to a thione tautomer in the solid state as it is observed in solution. Intermolecular hydrogen bond interactions are observed in the ligand with the imine group being the hydrogen bond donor to the adjacent sulphur atom of the next nearest molecule. The separation for this interaction, N2–H2⋯S1^i^, is determined to be 2.571 (6) Å (Figure 3).

**Figure 4:**
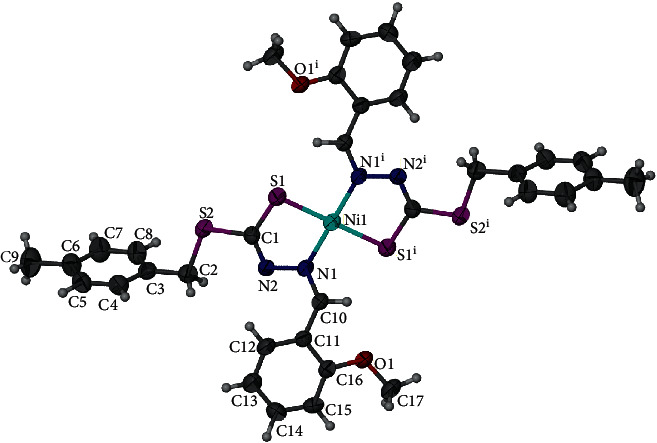
Structure of **3** with ellipsoids shown at 50% probability. Selected bond lengths [Å] and angles [°]: Ni1-N1 = 1.889 (6); Ni1–S1 = 2.153 (8); Ni-N2 = 1.393 (8); N1-Ni1-N1i = 180; and S1-Ni1-S1i = 180. Symmetry element used: *i* = 2−*x*, −*y*, 2−*z*.

**Figure 5:**
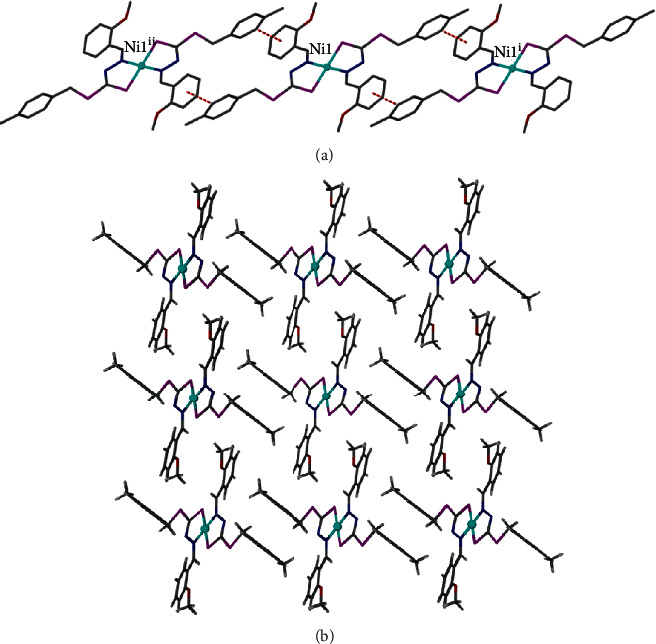
(a) *π*-*π* interactions between the neighbouring complexes. Symmetry elements used: ^i^ = 1 + *x*, *y*, *z*−1; ^ii^ = 1−*x*, −*y*, 3−*z*. (b) Interdigitating arrangement of the aromatic rings within **3**.

**3** crystallizes in triclinic space group *P*-1 with half of the molecule forming an asymmetric unit (Figure 4). The coordination sphere of the central Ni1 ion is occupied by two imine nitrogen and two sulphur atoms of two ligands, which are arranged in anti-conformation. The central Ni ion displays a square planar geometry with the geometry index, *τ* = 0. The distances of Ni1-N1 and Ni1–S1 are 1.889 (6) Å and 2.153 (8) Å, respectively, are comparable to the similar constituted complexes such as [Ni (L_3_) (py)] [Ni-N2 = 1.884 (7) Å, N1-S1 = 2.170 (3) Å] [58] and NiL_2_^1^ [N1–Ni1 = 1.9010 (2) Å, Ni1–S1 = 2.2015 (9) Å] [57]. These bond lengths are marginally shorter than the equivalent bonds in the oxomolybdenum(VI) [59], tin(IV) [60], and copper(II) [61] complexes of related dithiocarbazate ligands. In the crystal packing of **3**, the *π*-*π* interactions are the most dominant in stabilizing the structure with the closest separation being 3.823 (5) Å due to the lack of any potential hydrogen bond donor (Figure 5(a)). Further observation on the supramolecular *π*-*π* interactions within complex **3** has revealed that the aromatic moieties in complex **3** are arranged interdigitating to each other (Figure 5(b)).

### 3.3. Hirshfeld Surface Analysis

**Figure 6:**
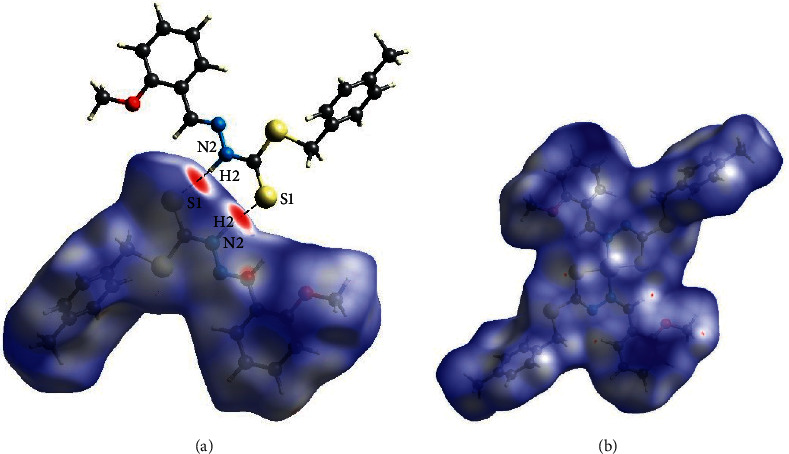
Hirshfeld surface mapped over *d*_norm_ for (a) **1** and (b) **3**.

The intermolecular interaction of crystal structures of **1** and **3** was quantified through the Hirshfeld surface analysis and 2D fingerprint plots using CrystalExplorer 17.5 [36]. The Hirshfeld surface mapped over *d*_norm_**1** and **3** is shown in Figures 6(a) and 6(b), respectively. There are bright-red spot regions in **1**, demonstrating the occurrence of a strong hydrogen bond connecting the neighbouring molecules via N2–H2⋯S1. However, no strong hydrogen bond interaction is visualized in **3**. From the 2D fingerprint plots (Figure S12), the greatest percentage contributions to the Hirshfeld surface for **1** (48.4%) and **3** (44.8%) are shown by the sharp spike characteristic at *d*_e_ *+* *d*_i_ ∼2.2 Å representing H⋯H contact. The H⋯C/C⋯H contact is the second most important contributor to intermolecular interactions, accounting for 23.6% for **1** and 26.4% for **3**, and is shown by a pair of peak characteristic at *d*_*e*_ *+* *d*_*i*_ ∼3.2 Å. These contacts may be due to the occurrence of C–H···*π* interactions in the compounds (Table 3). On the Hirshfeld surface mapped with the shape index, the corresponding C–H···*π* interactions (marked with circles in Figure S13) are observed for compounds **1** and **3**. The presence of intermolecular N–H⋯S interactions in **1** is corroborated by a pair of sharp peaks at *d*_*e*_ *+* *d*_*i*_ ∼ 3.0 Å, which constitute 15.9% of the H⋯S/S⋯H contact. The proportions of H⋯N/N⋯H, H ⋯ O/O⋯H, C ⋯ S/S⋯C, C ⋯ N/N⋯C, C ⋯ C, S ⋯N/N⋯S, C ⋯ O/O⋯C, and S⋯O/O⋯S interactions showed that an inferior contribution for **1** comprised 3.0%, 2.6%, 2.2%, 1.7%, 1.1%, 0.4%, 0.2%, and 0.1% of the Hirschfeld surface, respectively. In **3**, the weak contacts are attributed to H⋯N/N⋯H, H⋯O/O⋯H, H⋯Ni/Ni⋯H, C⋯S/S⋯C, C⋯C, S⋯N/N⋯S, C⋯N/N⋯C, and O⋯O that involved 3.7%, 3.2%, 2.3%, 1.8%, 1.3%, 1.3%, 0.4%, and 0.1%, respectively. Overall, these intermolecular interactions provide further stability in the crystal structure of both compounds [36].

### 3.4. Cytotoxic Activity

The cytotoxicity of studied compounds was tested against two breast cancer cell lines: MCF-7 and MDA-MB-231. With the exception of **2**, none of the other compounds tested inhibited both cell lines significantly. The GI_50_ value of 0.85 *μ*M and 0.37 *μ*M suggests that 2 is considered as strongly active against MDA-MB-231 and MCF-7 cancer cells, respectively as compared to cisplatin (GI_50_ = 12.0 *μ*M (MDA-MB-231) and 6.5 *μ*M (MCF-7)) [56]. Based on the data obtained, **2** is more sensitive to estrogen receptor-positive breast cancer (MCF-7) cells. This is where estrogen hormones play an essential role in treating cancers compared with triple-negative estrogen receptors (MDA-MB-231). Besides, **2** could induce chromatin condensation and nuclear fragmentation, suggesting the presence of apoptotic bodies of cells [7]. However, the complexation of the **1** with Cu(II) ions brings a significant decrease in the polarity of copper ions. The resulting increase in the lipophilic property of the Cu(II) ion brings an increase in the permeability of the Cu(II) complex across the lipid bilayer of the cell membrane [48]. In addition, the hydration energy release in aquatic media favours the reduction of Cu(II) to Cu(I), whereas Zn(II) and Ni(II) do not undergo this reduction [62]. As a result, complex **2** investigated in this experiment could be a promising anticancer therapeutic candidate that warrants further exploration into its mechanism of action.

### 3.5. DNA-Binding Studies

#### 3.5.1. Absorption Spectroscopic Studies

**Figure 7:**
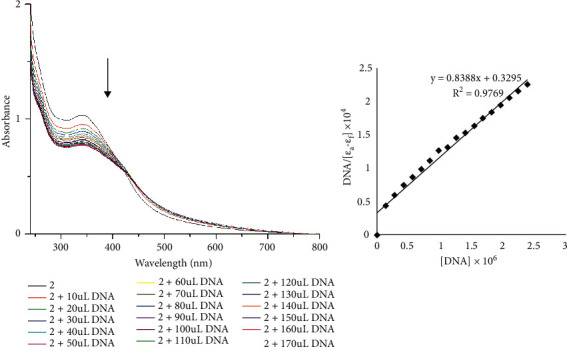
Electronic absorption spectrum and plot of [DNA]/*ε*_a_−*ε*_f_ vs. [DNA] for absorption titration of DNA with compound **2**.

UV-Vis absorption spectral titrations were performed to explore the interactions between cytoactive complex 2 and CT-DNA.  Figure 7 shows the absorption spectra of 2 in the absence and presence of CT-DNA as a function of concentration. Complex 2 exhibited a strong absorption peak at *λ*_max_ 342, corresponding to *π*⟶*π*^*∗*^ intra-ligand transition of the aromatic chromophore. The intensity of the prominent absorption was reduced as the concentration of CT-DNA was increased, and a bathochromic shift (1 to 5 nm) was observed in **2**. The changes reveal that the aromatic chromophore of 2 exhibits promising interactions with the aromatic base pairs of the DNA, suggesting a good binding with a 25.51% hypochromic shift. The intrinsic binding constant (*K*_b_) of 2 was 2.55 × 10^4^ M^−1^, which is consistent with previous reports showing intermediate binding interactions between complexes and CT-DNA [63]. The obtained *K*_b_ values were lower than those of the classical intercalator (ethidium-DNA, 3 × 10^6^ M^−1^) [64]. This indicates that 2 has a less binding affinity to the CT-DNA than ethidium bromide [63–65].

#### 3.5.2. Molecular Docking Simulation

**Figure 8:**
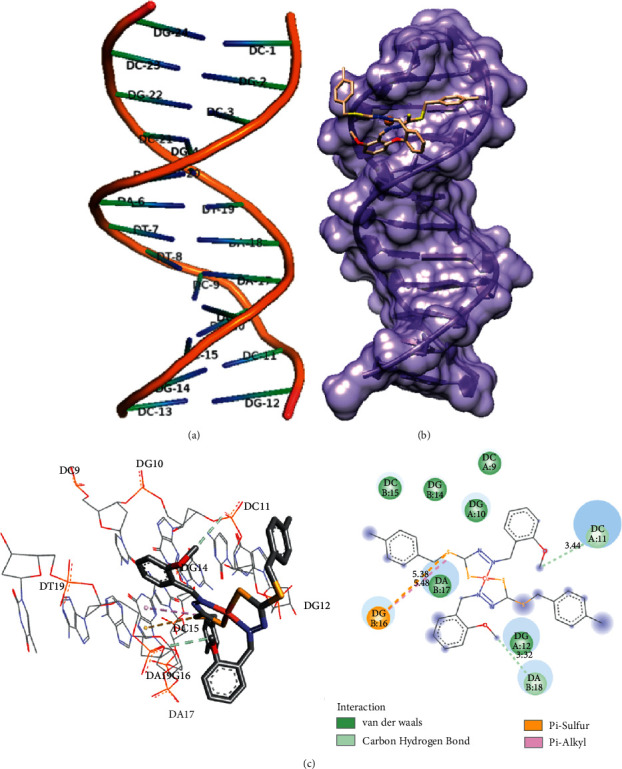
(a) DNA duplex of sequence (PDB ID : 1BNA) with labels. (b) The lowest binding energy conformation between **2** with DNA. (c) Molecular docked model showing the interactions of **2** with DNA.

Molecular docking simulation plays a significant role in the development of new chemotherapeutic drugs. It is used to understand the interactions between synthesized drugs and DNA, supporting the experimental investigation of DNA binding. The conformation of docked complex **2** was analysed based on binding energy and non-covalent (hydrogen bonding, major or minor groove, and electrostatic) interactions between complex **2** and DNA. The labelled DNA duplex of sequenced (CGCGAATTCGCG)_2_ dodecamer used is given in Figure 8(a). The molecular docking of **2** with DNA duplex yielded the most suitable docked poses (Figure 8(b)). As illustrated in Figure 8(c), we can express that the **2** fit closely into the G-C region of DNA via hydrogen bonding and hydrophobic and van der Waals interactions with binding energy −7.39 kcal/mol. This demonstrates the higher binding affinity in the G-C region compared with the adenine-thymine (AT)-rich region with a binding energy of −4.38 kcal/mol. The G-C region is crucial in DNA stability because three hydrogen bonds stabilize guanine and cytosine, and it has been suggested that targeting these regions could have a key role in anticancer activity [66, 67]. Complex **2** forms two carbon-hydrogen bonds between the C atom of complex **2** with phosphate groups of adenine-18 and cytosine-11 with the bond distance of 3.32 and 3.44 Å, respectively. The other non-covalent bonds between **2** and DNA bases are shown in Figure 8(c). Significant cytotoxicity was observed, although DNA-binding studies revealed that complex **2** did not strongly bind to DNA. The mechanism of action of complex **2** remains unknown and is a subject for future research.

## 4. Conclusions

A number of physicochemical studies and single-crystal X-ray diffraction analysis were performed to investigate the dithiocarbazate Schiff base ligand **1** and its complexes **2**, **3**, and **4**.

The in vitro cytotoxic activity of the synthesized compounds against MCF-7 and MDA-MB-231 cells was tested. However, only complex **2** demonstrated significant cytotoxic potency against both cancer cells. The absorbance and molecular docking simulations were used to further investigate the DNA-binding studies of **2**, which reveal that **2** binds to DNA rather effectively via hydrogen bonding and hydrophobic, and van der Waals interactions. The obtained data correlate well with the cytotoxicity of the complex, indicating that **2** has the potential to be a promising anticancer candidate.

## Figures and Tables

**Table 1:** Crystallographic data for **1** and **3**.

	**1**	**3**
Formula	C_17_H_18_N_2_OS_2_	C_34_H_34_N_4_NiO_2_S_4_
Formula weight	330.45	717.60
Crystal system	Monoclinic	Triclinic
Space group	P2_1_/*n*	P-1
*a* (Å)	5.4228 (11)	7.500 (7)
*b* (Å)	21.628 (4)	10.463 (10)
*c* (Å)	14.139 (3)	10.717 (10)
*α* (°)	90	93.700 (15)
*β* (°)	94.18 (3)	93.408 (16)
*γ* (°)	90	93.802 (17)
*V* (Å^3^)	1653.8 (6)	835.6 (14)
*Z*	4	1
Density calcd (g cm^−3^)	1.327	1.483
*F* (000)	696	374
*R*_int_	0.0242	0.0925
*R*_1_/*wR*_2_ (I > 2*σ* (*I*))	0.0357/0.0906	0.0513/0.0790
GooF	1.023	1.022

**Table 2:** Selected bond lengths and bond angles of **1** and **3**.

**1**			
Bond lengths (Å)		Bond angles (°)	

S2˗C2	1.8199 (18)	C1˗S2˗C2	101.93 (9)
S2˗C1	1.7564 (17)	N2˗C1˗S1	122.03 (13)
S1˗C1	1.6743 (18)	S1˗C1˗S2	124.82 (11)
N2˗C1	1.335 (2)	C1˗N2˗H2	120.8 (14)
N1˗C10	1.281 (2)	C1˗N2˗N1	119.05 (14)
N2˗N1	1.376 (2)	C10˗N1˗N2	116.63 (14)
C3˗C2	1.514 (2)		
**3**			
Ni1˗N1	1.889 (5)	N1˗Ni1˗N1	180.0
Ni1˗S1	2.153 (2)	N1˗Ni1˗S1	85.75 (19)
Ni1˗S1	2.153 (2)	N1˗Ni1˗S1	94.25 (19)
C1˗N2	1.277 (7)	S1˗Ni1˗S1	180.0
C1˗S1	1.698 (7)		
C1˗S21.727 (7)			

**Table 3:** Hydrogen bond geometry and intermolecular interactions (Å, °).

D˗H···A		D˗H	H···A	D···A	D˗H···A	
*Intermolecular hydrogen bond*						

**1**	N2˗H2⋯S1	0.85 (2)	2.57 (2)	3.4128 (17)	169.3 (18)	
	X˗H···*Cg*	X˗H	H···*Cg*	X···*Cg*	X˗H···*Cg*	Symmetry codes

*X˗H···π interactions*						
**1**	C14˗H14···*Cg*1		2.75	3.518 (2)	141	1−X, 1−Y, 1−Z
	C17˗H17C···*Cg*1		2.91	3.415 (2)	114	3/2−X, 1/2 + Y, 3/2−Z

**3**	C4˗H4···*Cg*1		2.73	3.537 (8)	143	−1 + X, Y, Z
	C4˗H4···*Cg*2		2.73	3.537 (8)	143	1−X, −Y, 2−Z
	C7˗H7···*Cg*4		2.89	3.833 (9)	172	1−X, −Y, 3−Z
	C17˗H17C···*Cg*3		2.81	3.639 (9)	142	1 + X, 1 + Y, Z

## Data Availability

CCDC 2106915 (**1**) and 2106916 (**3**) contain all crystallographic data and can be obtained free of charge via the Cambridge Crystallographic Data Centre.

## Supplementary Materials

Supplementary Materials Table S1. Experimental and calculated FTIR vibrations (cm^−1^) for the Schiff bases **1** and its Cu(II), Ni(II), and Zn(II) complexes (**2**–**4**). Table S2. Experimental and calculated electronic spectral data of the Schiff bases 1 and its Cu(II), N(II), and Zn(II) complexes (**2**–**4**). Figure S1. FTIR spectrum of 1. Figure S2. FTIR spectrum of 2. Figure S3. FTIR spectrum of 3. Figure S4. FTIR spectrum of 4. Figure S5. ^1^H NMR spectrum of 1. Figure S6. ^13^C NMR spectrum of 1. Figure S7. ^1^H NMR spectrum of 3. Figure S8. ^13^C NMR spectrum of 3. Figure S9. ^1^H NMR spectrum of 4. Figure S10. ^13^C NMR spectrum of 4. Figure S11. Mass spectrum of Schiff base 1. Figure S12. Fingerprint plots of all H⋯H, H⋯C/C⋯H, and H⋯S/S⋯H interactions, showing the percentage of contacts contribute to the total Hirshfeld surface area of (a) **1** and (b) **3**. Figure S13. Hirshfeld surface of compounds (a) **1** and (b) **3** plotted over shape index.
