# Supplementary material for: Dithiocarbazate Ligand-Based Cu(II), Ni(II), and Zn(II) Complexes: Synthesis, Structural Investigations, Cytotoxicity, DNA Binding, and Molecular Docking Studies
Source: Bioinorg Chem Appl. 2022 Jul 31;2022:2004052. doi: 10.1155/2022/2004052 (PMC9357781; doi:10.1155/2022/2004052)
Supplement: Supplementary Materials — Table S1. Experimental and calculated FTIR vibrations (cm−1) for the Schiff bases 1 and its Cu(II), Ni(II), and Zn(II) complexes (2–4). Table S2. Experimental and calculated electronic spectral data of the Schiff bases 1 and its Cu(II), N(II), and Zn(II) complexes (2–4). Figure S1. FTIR spectrum of 1. Figure S2. FTIR spectrum of 2. Figure S3. FTIR spectrum of 3. Figure S4. FTIR spectrum of 4. Figure S5. 1H NMR spectrum of 1. Figure S6. 13C NMR spectrum of 1. Figure S7. 1H NMR spectrum of 3. Figure S8. 13C NMR spectrum of 3. Figure S9. 1H NMR spectrum of 4. Figure S10. 13C NMR spectrum of 4. Figure S11. Mass spectrum of Schiff base 1. Figure S12. Fingerprint plots of all H⋯H, H⋯C/C⋯H, and H⋯S/S⋯H interactions, showing the percentage of contacts contribute to the total Hirshfeld surface area of (a) 1 and (b) 3. Figure S13. Hirshfeld surface of compounds (a) 1 and (b) 3 plotted over shape index. [file 2004052.f1.docx]

**SUPPLEMENTARY INFORMATION**

**Dithiocarbazate ligand based Cu(II), Ni(II) and, Zn(II) complexes: Synthesis, Structural Investigations, Cytotoxicity, DNA Binding and Molecular Docking Studies**

Enis N. M. Yusof^1*^, Mohammad Azam^2^, Siti S. Sirat^3^, Thahira B. S. A. Ravoof^4^, Alister J. Page^5^, Abhi Veerakumarasivam^6,7^, Thiruventhan Karunakaran^8,9^, Mohd R. Razali^8^

*^1^Chemistry Section, School of Distance Education, Universiti Sains Malaysia, 11800, Minden, Penang, Malaysia*

*^2^Department of Chemistry, College of Science, King Saud University, PO BOX 2455, Riyadh 11451, Saudi Arabia*

*^3^Faculty of Applied Sciences, Universiti Teknologi MARA, Cawangan Negeri Sembilan, Kampus Kuala Pilah, 72000 Kuala Pilah, Negeri Sembilan, Malaysia*

*^3^Department of Chemistry, College of Science, King Saud University, PO BOX 2455, Riyadh 11451, Saudi Arabia*

*^4^Department of Chemistry, Faculty of Science, Universiti Putra Malaysia, 43400 UPM Serdang, Selangor, Malaysia.*

*^5^Discipline of Chemistry, School of Environmental and Life Sciences, University of Newcastle, University Drive, Callaghan, NSW, 2308, Australia.*

*^6^Department of Biological Sciences, School of Medical and Life Sciences, Sunway University, No. 5 Jalan Universiti, 47500 Bandar Sunway, Selangor Darul Ehsan, Malaysia*

*^7^Medical Genetics Laboratory, Faculty of Medicine and Health Sciences, Universiti Putra Malaysia, 43400 UPM Serdang, Selangor Darul Ehsan, Malaysia*

*^8^Centre for Drug Research, Universiti Sains Malaysia, 11800, Minden, Pulau Pinang, Malaysia*

*^9^School of Chemical Sciences, Universiti Sains Malaysia, 11800 USM, Pulau Pinang, Malaysia*

*****Correspondence: [enisnadia@usm.my](mailto:enisnadia@usm.my)

**Table S1.** Experimental and calculated FTIR vibrations (cm^-1^) for the Schiff bases **1** and its Cu(II), Ni(II) and Zn(II) complexes (**2**-**4**).

| Compound | Method | IR bands (cm^-1^) | | | |
| --- | --- | --- | --- | --- | --- |
|  |  | *v*(NH) | *v*(C=N) | *v*(N-N) | *v*(C=S)/  *v*(C-S) |
| 1 | Experimental | 3105 | 1600 | 1153 | 948 |
|  | B3LYP/6-311G(d,p) | 3428 | 1607 | 1132 | 942 |
| 2 | Experimental | - | 1596 | 1100 | 943 |
|  | B3LYP/LanL2DZ/6-311G(d,p) | - | 1612 | 1035 | 944 |
| 3 | Experimental | - | 1589 | 1107 | 964 |
|  | B3LYP/6-311G(d,p) | - | 1611 | 1028 | 969 |
| 4 | Experimental | - | 1585 | 1100 | 944 |
|  | B3LYP/LanL2DZ/6-311G(d,p) | - | 1636 | 1054 | 958 |

**Fig. S1 FTIR spectrum of 1**

**Fig. S2 FTIR spectrum of 2**

**Fig. S3 FTIR spectrum of 3**

**Fig. S4 FTIR spectrum of 4**


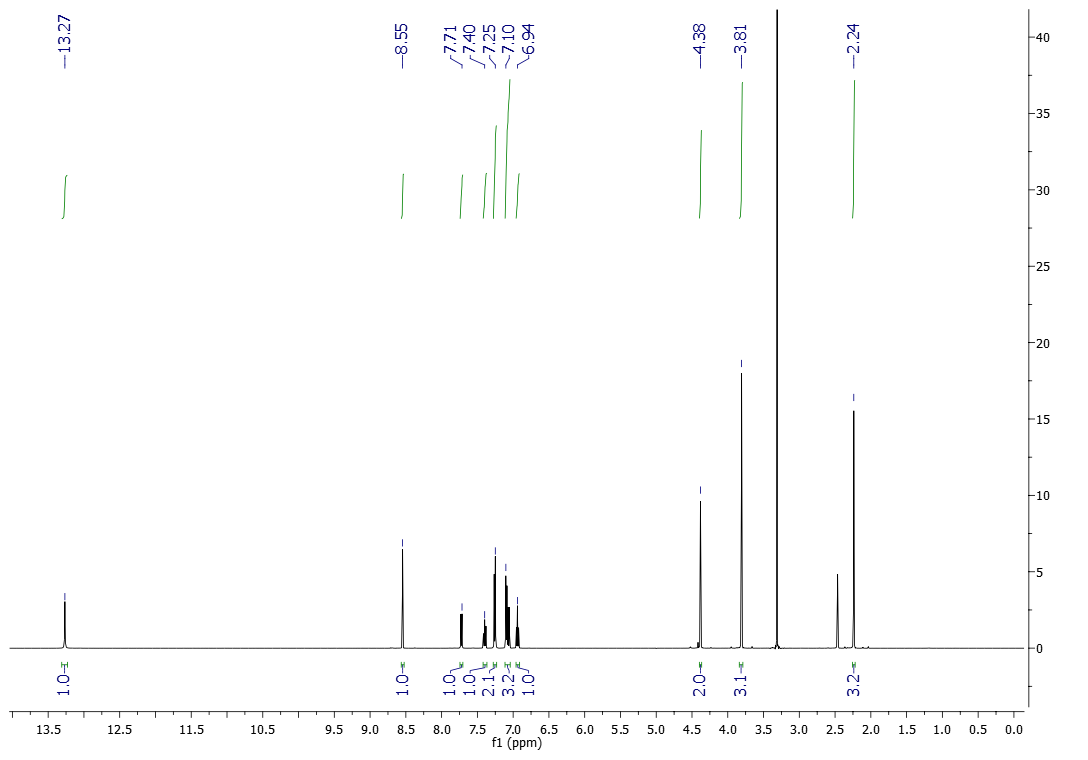


**Fig. S5 ^1^H NMR spectrum of 1**


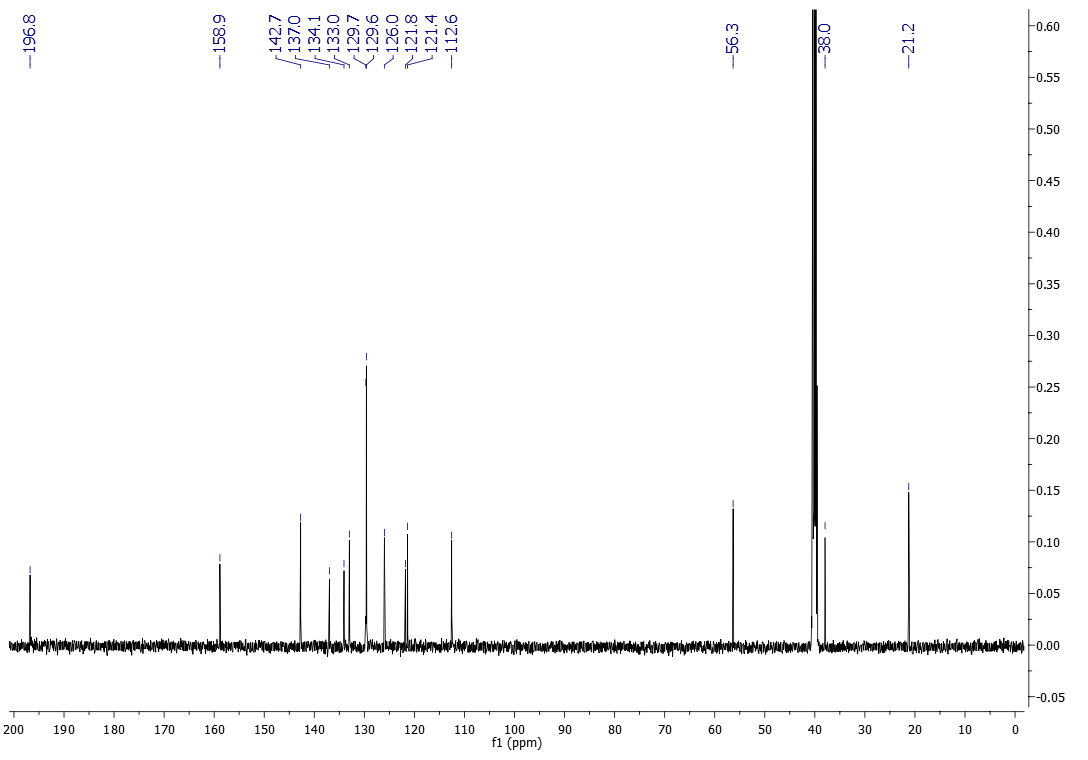


**Fig. S6 ^13^C NMR spectrum of 1**

**
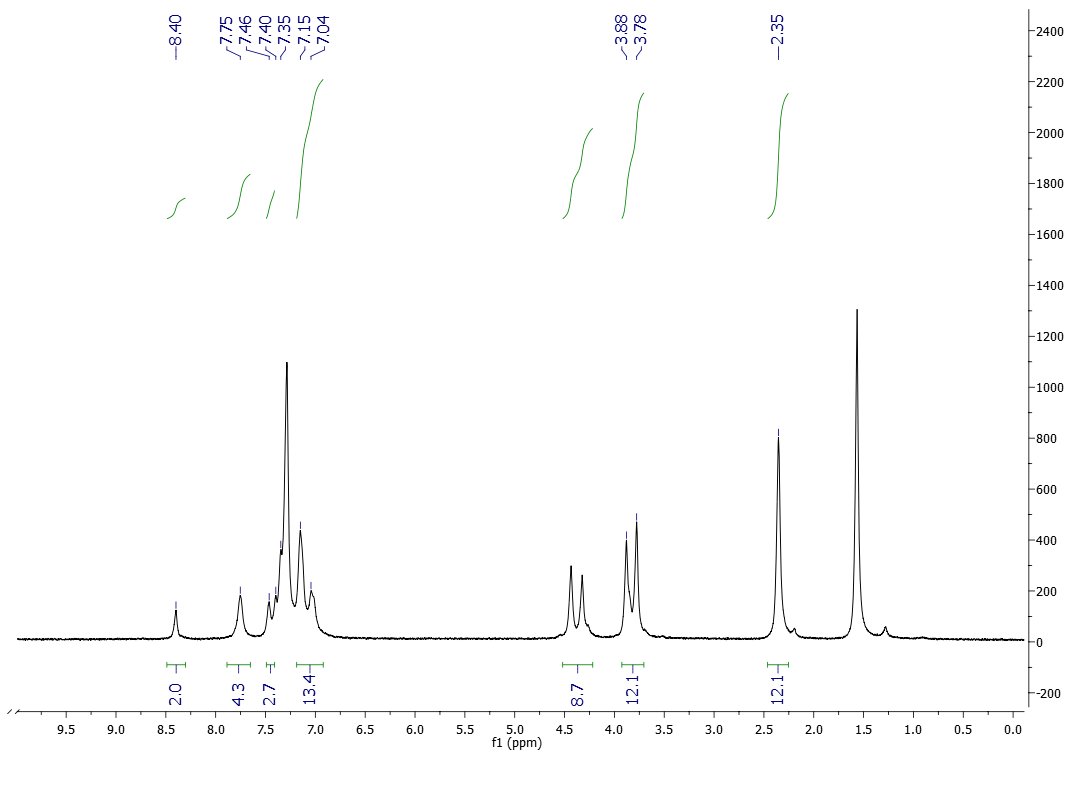
**

**Fig. S7 ^1^H NMR spectrum of 3**

**
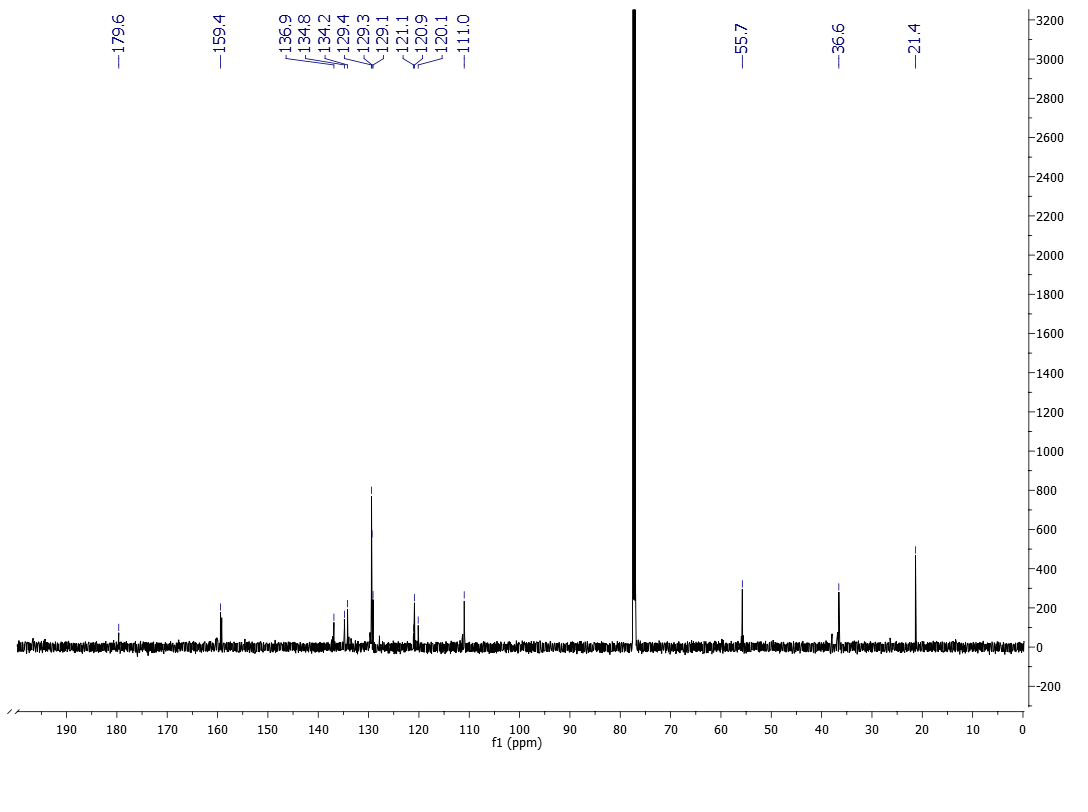
**

**Fig. S8 ^13^C NMR spectrum of 3**

**
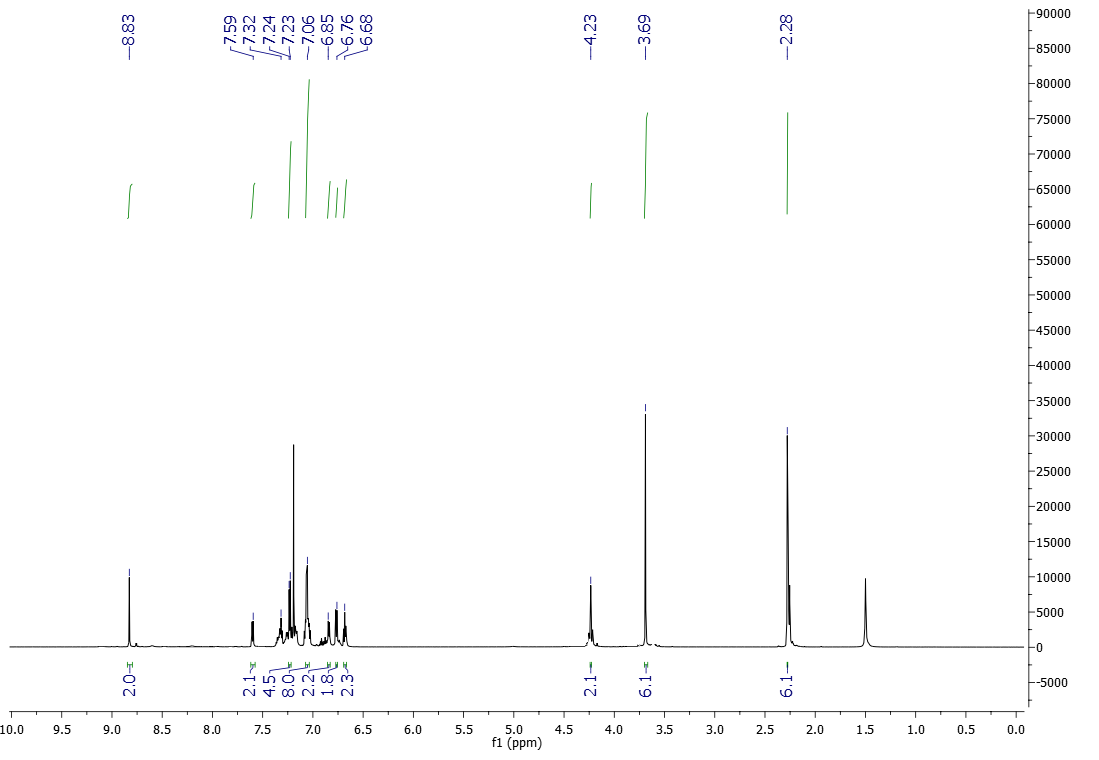
**

**Fig. S9 ^1^H NMR spectrum of 4**

**
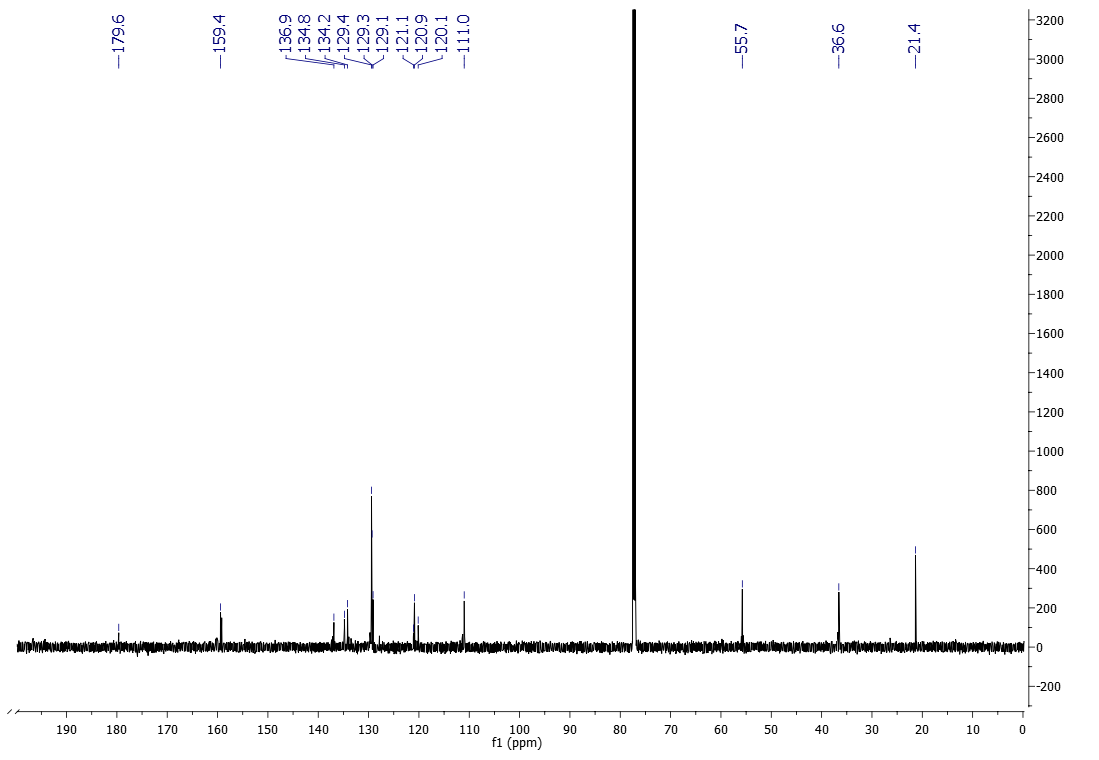
**

**Fig. S10 ^13^C NMR spectrum of 4**

**
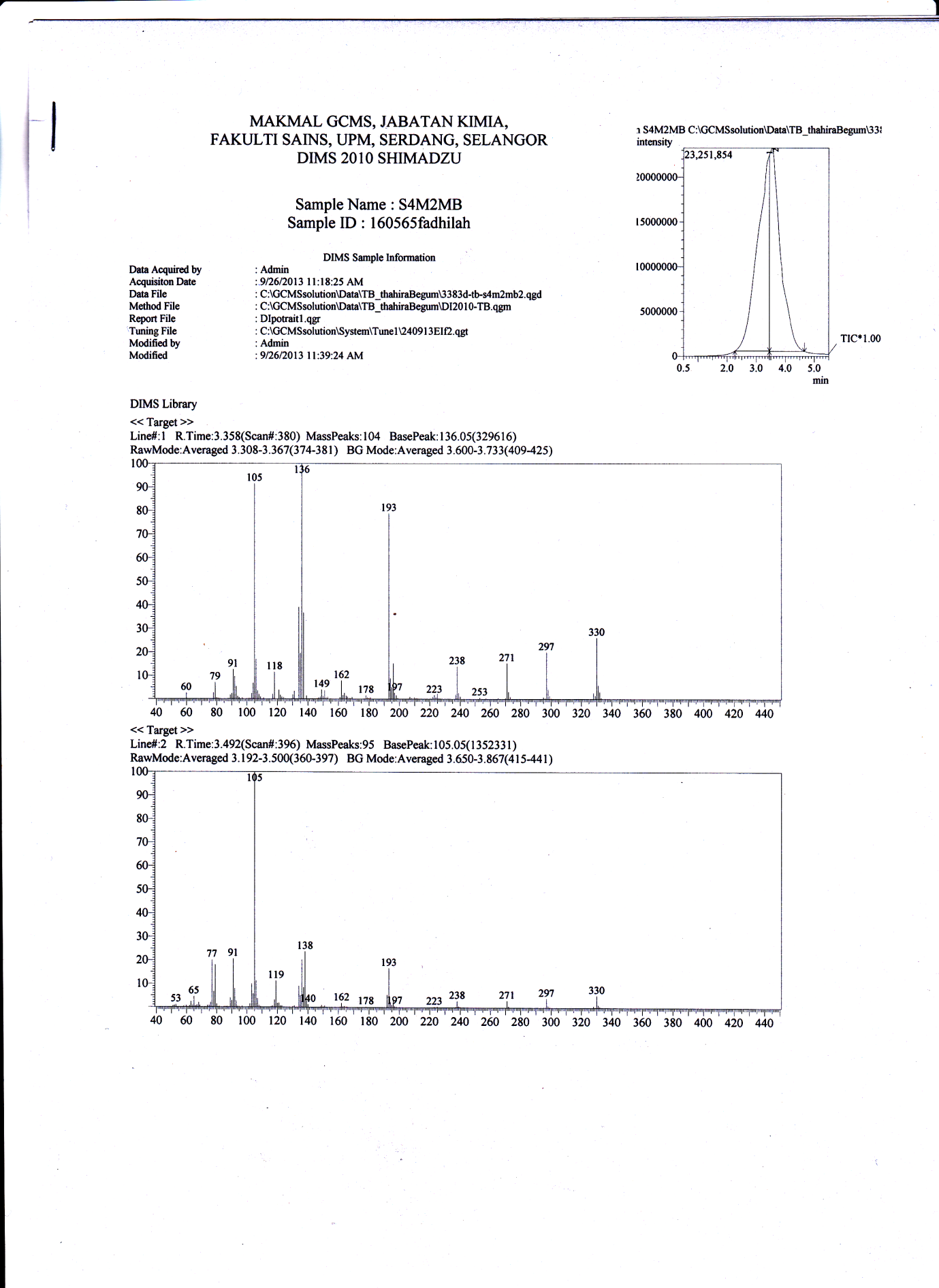
**

**Fig. S11** Mass spectrum of Schiff base **1**

**Table S2.** Experimental and calculated electronic spectral data of the Schiff bases **1** and its Cu(II), Ni(II) and Zn(II) complexes (**2**-**4**).

| Compound | Wavelength (nm) | |
| --- | --- | --- |
|  | Experimental (log ε_max_) | B3LYP/6-311G(d,p) or  B3LYP/LanLD2Z/6-311G(d,p) |
| **1** | 356(4.73) | 348 |
| **2** | 332(4.511); 515(0.85) | 477; 531 |
| **3** | 356(4.97); 369(4.86); 600(1.96) | 371; 416; 425 |
| **4** | 353(5.04) | 354; 376 |


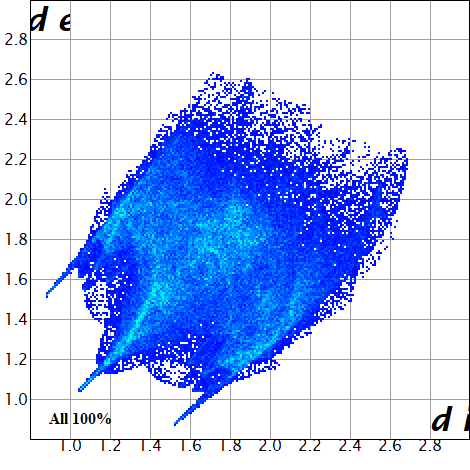

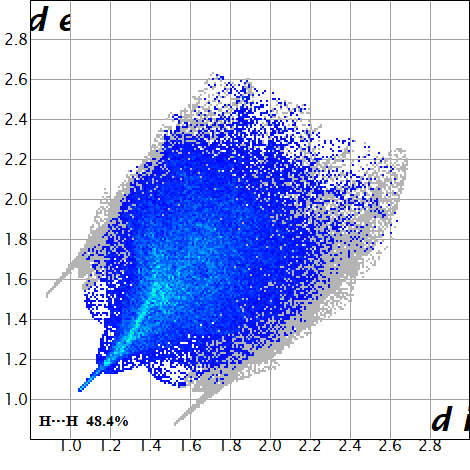

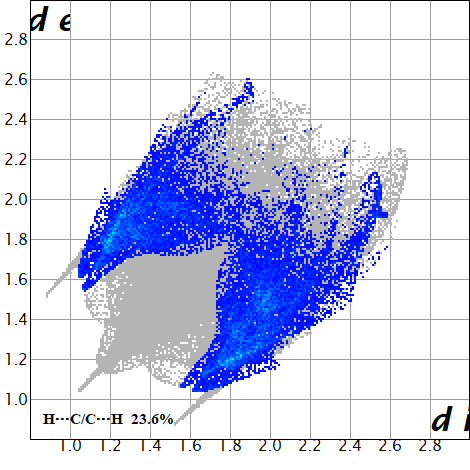

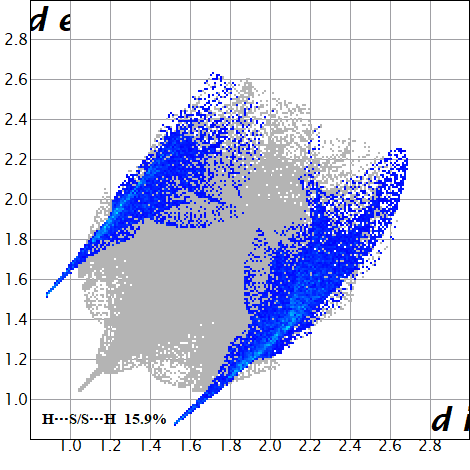


(a)


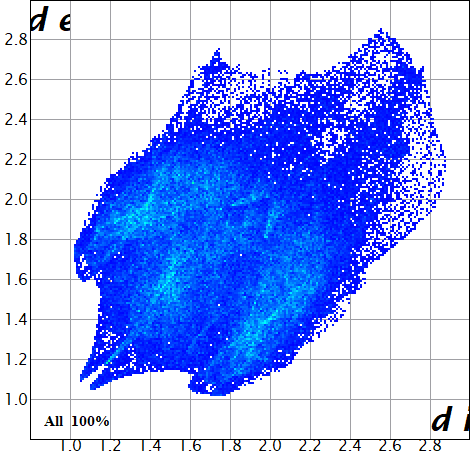

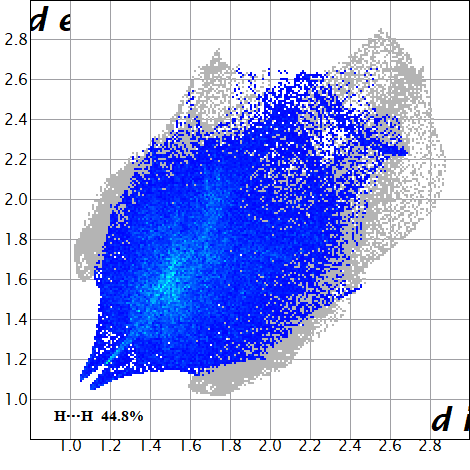

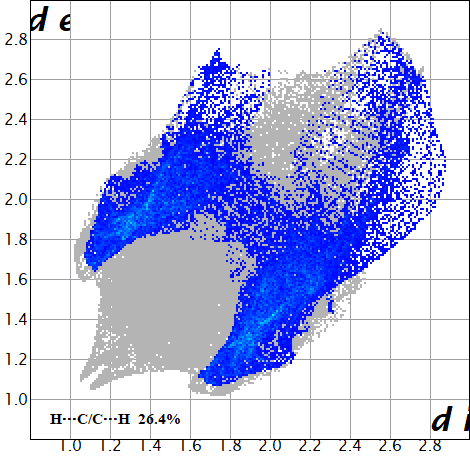

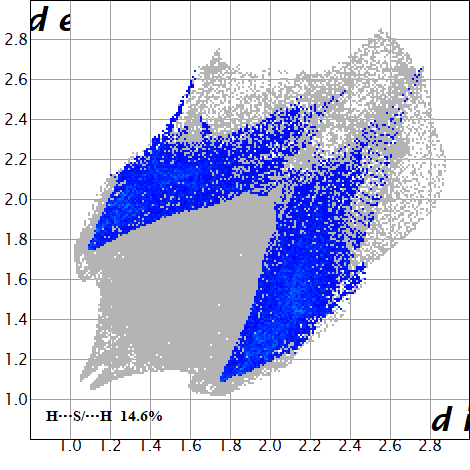


(b)

**Fig. S12** Fingerprint plots of all, H⋯H, H⋯C/C⋯H and H⋯S/S⋯H interactions, showing the percentage of contacts contribute to the total Hirshfeld surface area of (a) **1** and (b) **3.**


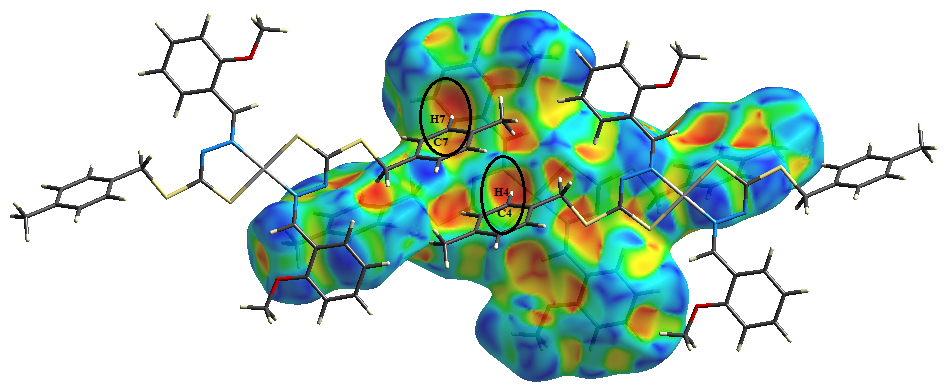

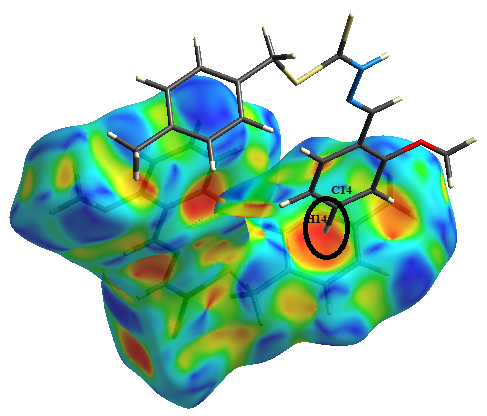
 (a)
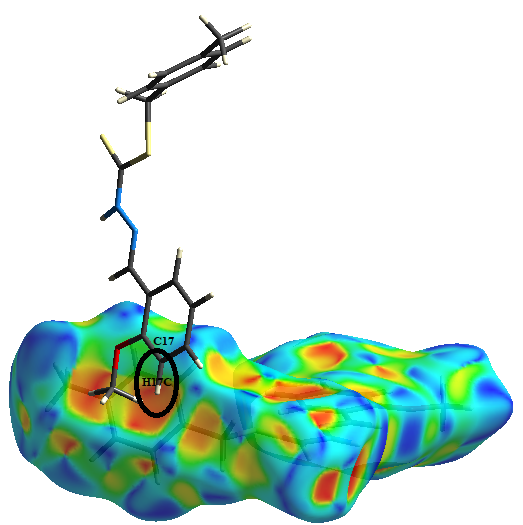


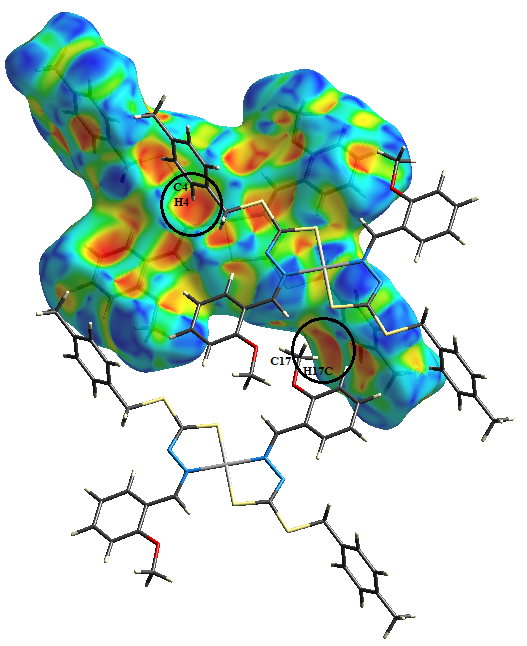


(b)

**Fig. S13** Hirshfeld surface of compound (a) **1** and (b) **3** plotted over shape-index.
